# Supplementary figures and images for: Genome-Wide Association Study (GWAS) for resistance to Sclerotinia sclerotiorum in Common Bean
Source: Genes (Basel). 2020 Dec 12;11(12):1496. doi: 10.3390/genes11121496 (PMC7764677; doi:10.3390/genes11121496)

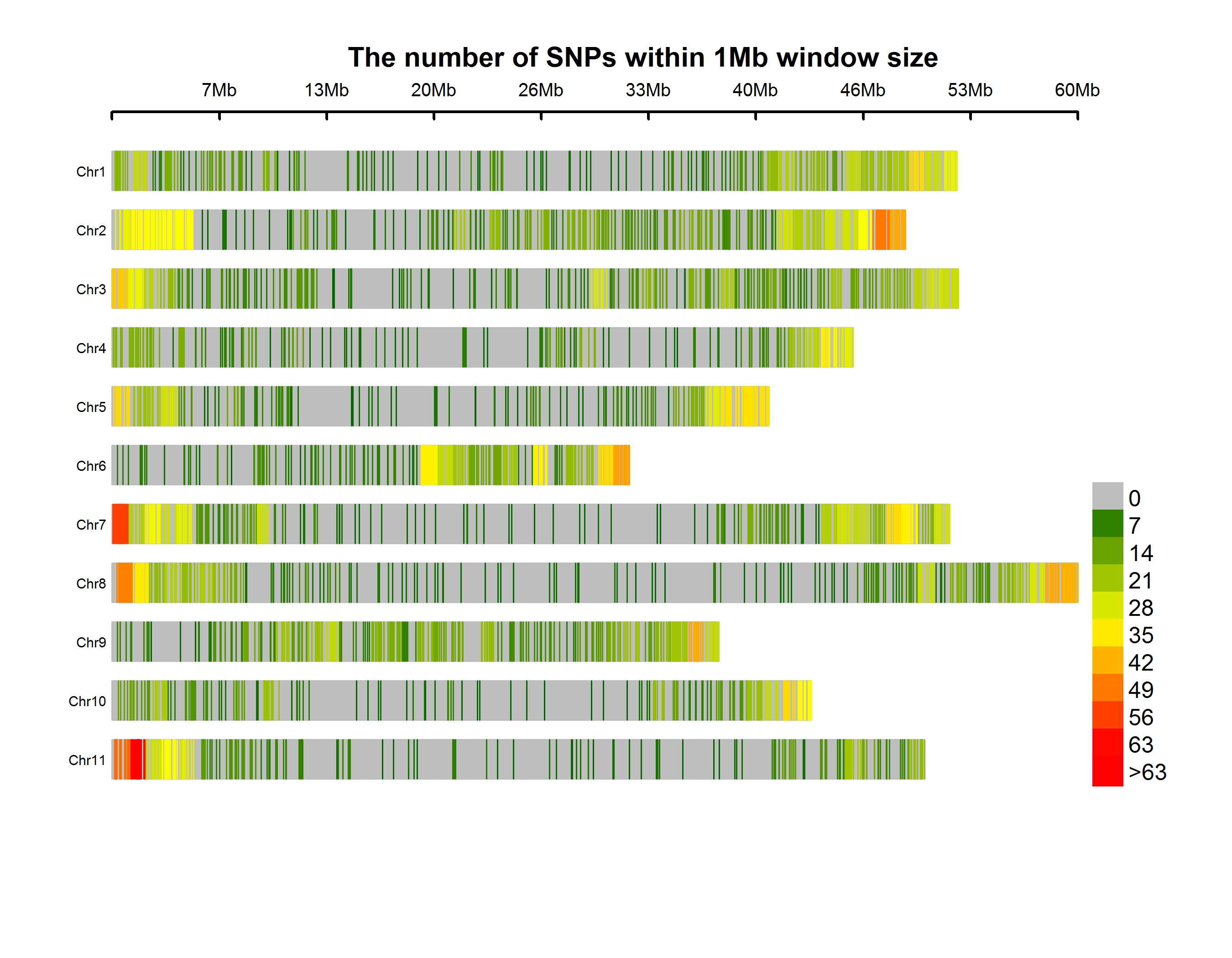

Supplement: Supplementary file 1 [file genes-11-01496-s001.zip › FigureS1.jpg]

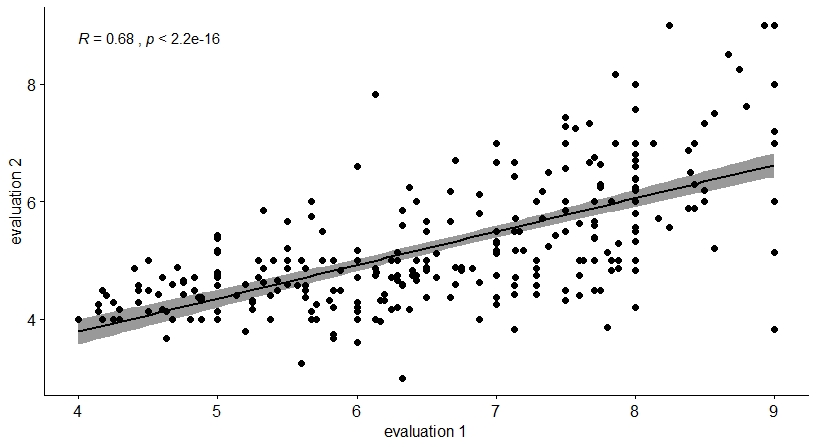

Supplement: Supplementary file 1 [file genes-11-01496-s001.zip › FigureS2.jpeg]
